# Testing Different Deterrents as Candidates for Short-Term Reduction in Wild Boar Contacts—A Pilot Study

**DOI:** 10.3390/ani10112156

**Published:** 2020-11-19

**Authors:** Nicolai Denzin, Frithjof Helmstädt, Carolina Probst, Franz J. Conraths

**Affiliations:** 1 Friedrich-Loeffler-Institut, Institute of Epidemiology, 17493 Greifswald, Germany; Carolina.Probst@fli.de (C.P.); Franz.Conraths@fli.de (F.J.C.); 2 Fachrichtung Forstwissenschaften, Technische Universität Dresden, 01737 Tharandt, Germany; f.helmstaedt@gmx.net

**Keywords:** African swine fever, wild boar, deterrent, carcass, habitat cycle, endemic

## Abstract

**Simple Summary:**

African swine fever is an important pig disease currently present in the wild boar population, in particular in parts of Europe, with occasional introductions into domestic pig farms. Lately, the first cases were detected in wild boar in Eastern Germany. The presence of the disease dramatically affects the chances of a country to participate in international trade with pigs and products thereof. Limiting disease spread with the goal of eventual eradication is therefore of paramount importance. Carcasses of wild boar that succumbed to African swine fever represent an important source of infection and support the perpetuation of the infection cycle. Hence, timely removal of carcasses from the environment in infected areas is an important disease control measure but is sometimes difficult due to logistic limitations—e.g., in forests or thickets. Deterring wild boar from carcasses may therefore constitute an interim solution. We aimed at identifying suitable deterrence strategies and found that certain chemical and physical deterrents seem to deter wild boar, to some extent, are easy to apply and may thus contribute to disease control. In depth investigation of the deterrence effect of the promising deterrent candidates identified in this pilot study should be considered.

**Abstract:**

African swine fever (ASF) is a viral infection of pigs and represents a major threat to animal health and trade. Due to the high tenacity of the causative virus in carcasses of wild boar, contacts of wild boar with infectious carcasses are regarded an important driver of the so-called habitat cycle. The latter is believed to play a major role in maintaining the present ASF situation in wild boar in Europe. Therefore, search campaigns and timely removal and disposal of carcasses are considered important disease control approaches. If timely disposal is not feasible due to logistic reasons, deterrence of wild boar may be a provisionary option. The performance of seven deterrents (physical and chemical) was tested in a forest near Greifswald, Germany. Carcasses as entities of attraction for wild boar were substituted by luring sites. It could be demonstrated in this pilot study that certain physical (LED blinkers, aluminum strips) and chemical (HAGOPUR Wildschwein-Stopp™, Hukinol™) deterrents are capable of reducing the odds of wild boar contacts to one third, but in depth testing of the aforementioned promising deterrent candidates is recommended. A choice of those deterrents identified as suitable, reasonable, and easy to apply should be carried out, when carcass search campaigns are launched in the case of an outbreak of ASF in wild boar.

## 1. Introduction

African swine fever (ASF) is a viral infection of pigs and a major threat to animal health and trade. Since the detection of the first ASF case in wild boar in Lithuania in late January 2014, more than 27,000 cases in wild boar (*Sus scrofa*) have been registered in the Animal Health Notification System of the European Union. In September 2020 the first cases were confirmed in the German federal state of Brandenburg close to the border with Poland. In affected regions, a substantial number of wild boar die from infection, thus becoming available to invertebrate decomposers, vertebrate scavengers and susceptible conspecifics [1]. African swine fever virus (ASFV) is extremely stable in the environment and efficiently transmitted via blood and meat of infected animals. It can persist at 4 °C for over a year in blood, several months in boned meat and several years in frozen carcasses [2,3]. The virus also survives the process of putrefaction [4]. Due to its high tenacity, the spread of ASFV through carcasses is considered to be more important than direct contact with live infectious animals, depending on the frequency, at which naive animals have contact with infected carcasses within their range of daily movements [4,5,6,7]. It was shown that wild boar occasionally sniff and poke on carcasses (without leaving any signs of cannibalism—e.g., bite marks), chew on bare ribs and root on the soft soil that has formed after decomposition [8]. Both, direct transmission between wild boar, and indirect transmission via the habitat (carcasses and contaminated habitat) are the drivers of the recently described wild boar–habitat cycle [9] that determines the epidemiology of the current ASF epidemic in Central and Eastern Europe. The habitat contamination through ASFV-infected wild boar carcasses offers possibilities for new infections depending on landscape, time, season and carcass decomposition [8]. 

Both, the high tenacity of ASF virus and the long-time wild boar carcasses can remain in the environment and allow the persistence of the virus for several months or even years. Therefore, rapid detection and removal (or destruction on the spot) of contaminated carcasses are considered as an important control measure against ASF in wild boar [8].

If carcasses cannot be removed or buried immediately—e.g., for logistic reasons (difficult terrain, limited man power, additional technical equipment such as special vehicles required etc.)—the probability of effective (concerning infection) wild boar-carcass contact may, in the meantime, be reduced by application of deterrents to the carcasses. There is no information available on how frequently this strategy was already applied, but there were inquiries by disease control officials in the present epidemic in Germany concerning suitable deterrents for this purpose and the study was requested by German veterinary authorities. 

The aim of the presented study was to evaluate the suitability of different physical and chemical deterrents for short-term reduction in wild boar-carcass contacts. The examined deterrents comprised commercially available products marketed specifically for the protection of crops and gardens against, among other wildlife, wild boar as well as unspecific approaches. 

## 2. Materials and Methods

Since the study involved only standard luring techniques routinely applied in wildlife management and non-invasive, harmless deterrents, no ethical approval was necessary.

### 2.1. Study Area and Sites

**Figure 1:**
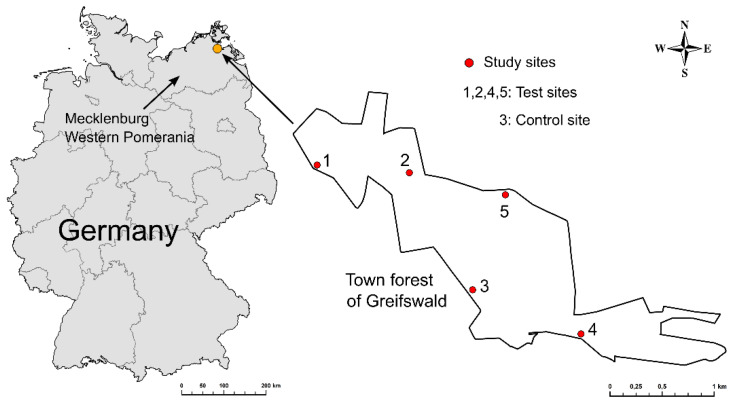
Location of the study sites in the city forest outside the town of Greifswald/Germany.

The study was carried out in the so-called town forest (though situated outside the city) of Greifswald, located at the Baltic Sea in the Federal State of Mecklenburg Western Pomerania, Germany. The study sites were placed next to frequently used wild boar trails known to the forest rangers in charge of clearings without underwood. Distances between sites within the forest were maximized to 900–1200 m in order to avoid interference. Five comparable sites were available based on estimated wild boar abundance, habitat (type and density of vegetation) and the abovementioned maximization of distances. Four sites were employed as test sites and one as a control site (Figure 1). The test sites were used twice to test all deterrent candidates.

**Figure 2:**
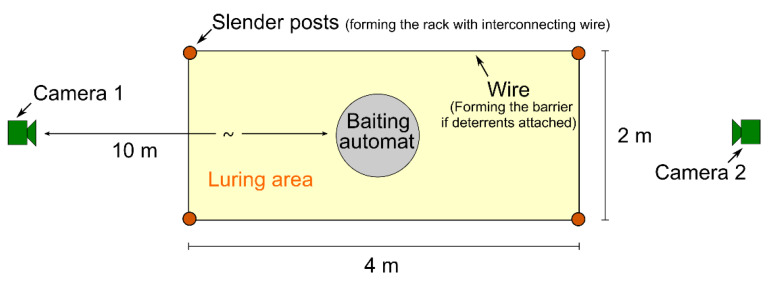
Standardized setup of the study sites.

Major attention was paid to the standardization of the sites. The carcasses as a potential entity of attraction to wild boar were substituted by a small luring area equipped with a baiting automat “WGI Futterautomat Quick Set™ 270” [10] (NewRoads, LA, USA). The luring area was bordered by a rectangle (2 × 4 m, see Figure 2) comprising four slender posts interconnected by a slim wire at a height of 1.6 m. The latter served as a rack to carry the deterrents under test (blank for the control site). The rectangle also defined the decision border with respect to the discrimination between success and failure of deterrence. Each site was monitored by two wildlife cameras facing towards each other at a distance of 10 m from the baiting automat. The cameras were fixed to massive wood trunks driven into the ground rather than to available trees to assure exactly the same layout of the sites.

### 2.2. Camera System

Seissiger “Special-Cam Classic HD 12 MP” wildlife cameras [11] (Würzburg, Germany) were used to monitor presence and behavior of wild boar throughout the study. The shutter lag was set to 30 s during baiting and “Wash-Out” phase and zero seconds during the study phases. 

### 2.3. Deterrent Systems

The deterrents tested comprised physical and chemical methods as well as combinations thereof. As physical approaches barrier tape (red and white; “Super Absperrband extrem reißfest”; [12], Jena, Germany), aluminum strips (as provided with the commercial product/kit “Wildschwein-Stopp”; [13], Landsberg, Germany) and commercially available battery-powered LED blinkers (blue; “Isotronic” [14], Oberndorf, Germany) were tested. The chemical methods employed were toilet deodorant blocks “WC FRISCH Kraft aktiv lemon” [15] (Düsseldorf, Germany), “Wildgranix™” [16] (Pommelsbrunn, Germany), “Hukinol™” [17], (Gottmadingen, Germany) and “Wildschwein-Stopp™” in combination with the aluminum strips included in the product kit.

#### 2.3.1. Physical Deterrents

##### 2.3.1.1. Aluminum Strips

The strips had a size of 10 × 30 cm and were accordion-folded. They were fixed to the wire of the rack via a cord of 10 cm length with rotation swivel. Three strips were attached to each long and two to each short side of the rack, respectively.

##### 2.3.1.2. LED Blinkers

The LED blinkers had a dimension of 12 × 7 cm with a reflector area (“cat’s eye”, blue) of 5 × 7 cm. At night and during twilight hours the device also emits a light sequence of three flashes in three consecutive seconds per min via two blue light emitting diodes (LEDs), pausing for the rest of the min. One LED blinker was fixed to the wire of each side of the rack (blinkers were not synchronized).

##### 2.3.1.3. Barrier Tape

The barrier tape had a width of 8 cm and was striped in red and white. Strips of 60 cm length each were knotted to the wire of the rack so that the two ends were hanging from the wire with an equal length of about 30 cm. Six strips were attached to each long side and three to each short side of the rack, respectively.

#### 2.3.2. Chemical Deterrents

##### Toilet Blocks

Toilet blocks of the brand “WC FRISCH Kraft aktiv lemon”, which come with a mount to hang them into a water closet, were used. One mount was fixed to the wire on each side of the rack. 

##### Wildgranix™

Wildgranix™ is a deterrent promoted for use specifically against wild boar, but also against other animals. The finely milled dolomite brick is granulated with an additive of natural adjuvant and is encased in nature-identical odorous substances (for details, see the respective data sheet [18]). One hundred grams of granulate, wrapped in a cloth, were fixed to the wire on each side of the rack. 

##### Wildschwein-Stopp™

Wildschwein (WS)-Stopp™ is marketed specifically for wild boar deterrence. It comprises two components (A and B in different spray cans), which are sprayed on felt pads of the aluminum strips as described under Section 2.3.1.1. The product contains a variety of ingredients (e.g., isobutane and 3-methyl butyric acid; for details consult the respective data sheet [19]). One aluminum strip treated with the product was fixed to the wire on each side of the rack. 

##### Hukinol™

Hukinol™ is a liquid wildlife deterrent that contains isovaleric acid and 2-methyl butyric acid [20]. Four cloths were soaked with 20 mL of Hukinol™ and each and one was fixed to the wire on each side of the rack. 

### 2.4. Study Design

At first, wild boar were attracted to the experimental sites during a fifteen weeks baiting phase and the acceptance of the sites was monitored. In that phase, 650 g of corn were fed twice a day (at 7 pm and 1 am) by spreading it over an area of approximately 16 m^2^ inside and outside the luring area (as defined by the rack, Figure 2). During the study phase (4 weeks), the feeding was focused on a small area of about one m^2^ directly under the baiting automat in the center of the luring area. In the first study phase, only physical deterrents were used to avoid any potential carryover of residues to the second study phase, in which chemicals were tested. Site 1 was left blank in phase one, after almost no wild boar had shown up at this site. During the “Wash-Out” (4 weeks) between the study phases, the baiting regime was switched back to the widespread corn distribution as executed in the initial baiting phase. This phase was meant to re-attract wild boar to the site, in case there had been an effect of deterrence in phase I—i.e., to “wash out” memories of this deterrence in the wild boar. Luring at the control site followed exactly the same regime.

The study sites were visited every second week to check their integrity, refill feedstuff and change memory cards of the cameras. Maintenance was carried out in the same standardized fashion for all sites.

### 2.5. Data Management and Analysis

Images from memory cards were analyzed with the help of the software FFM 2.0 (Forstzoologie, TU Dresden, Germany [21]). A wild boar camera event was defined as a sequence of images showing wild boar taken by a single camera with no lag between the images of five minutes or more. For each event the maximum number of individual juvenile (one year of age and younger) and adult wild boar present at the site and the respective numbers of barrier breaches were recorded. Failure of deterrence (i.e., barrier breach) was assumed, if an individual had entered the luring area or at least passed the barrier (Figure 2) with its head at minimum once during an event. Events of the two cameras with overlapping time intervals (either only two camera events of the two cameras or a cascade of alternately overlapping camera events) were merged to single, combined events using the R package lubridate [22] in the open source software R (R Foundation for Statistical Computing, Vienna, Austria [23]). Confidence limits (according to Clopper-Pearson [24]) of the proportion of barrier breaches for the two age classes and different study sites and deterrents were calculated employing the R package binom [25]. In order to control for the potential confounder age class [26], pooled odds ratios (odds of barrier breach on test versus control site) and their confidence intervals were calculated applying the method of Mantel-Haenszel [27] as implemented in the R package fmsb [28].

## 3. Results

### 3.1. General Findings

During the entire study period (27 weeks, Table 1), 601 wild boar events with 1909 wild boar were detected at the five study sites. The distribution of event duration was markedly skewed to the right with a maximum of 57 min, a minimum of zero s (only one image with wild boars per event), a mean of 159 s and a median of 33 s. The number of individual boars identified in an event ranged from 1 to 24 with a mean of 3 and a median of 1 (distribution also skewed to the right). Additionally, a variety of other species had triggered camera shots with predators (fox, badger, raccoon, raccoon dog, marten) and birds (predominantly pigeons and ravens) causing the majority of events. The reasons for about one third of the events remained unknown (no animals visible on the images).

During the weeks of testing (Table 1, 8 weeks) 221 events with 683 wild boar (427 juveniles, 256 adults) were recorded at the five sites (Table 2).

### 3.2. Effect of Deterrents

#### 3.2.1. Effect of Physical Deterrents

**Figure 3:**
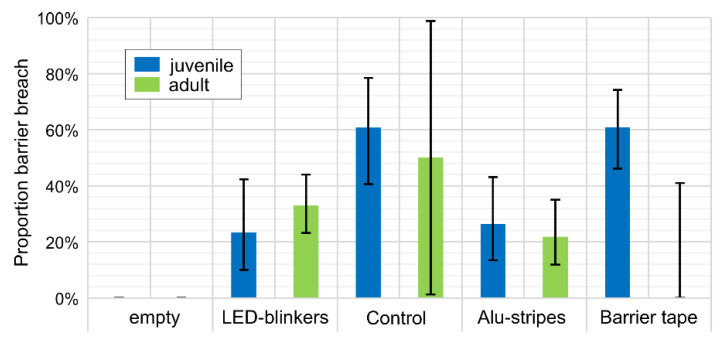
Proportion of barrier breaches with physical deterrence and 95% confidence intervals.

The effect of the physical deterrents is illustrated in Figure 3. Since study site 1 was only frequented by very few wild boar prior to testing (Table 1 and Table 2) the site was left blank. As compared to the control site, relatively few wild boar passed the barrier, when the LED blinkers and aluminum strips were in place. Barrier tape seemed to have no effect, at least not with respect to juvenile animals. Generally, juvenile wild boar seemed to be more prone to pass the barrier than adults, except in the experiment with LED blinkers.

#### 3.2.2. Effect of Chemical Deterrents

**Figure 4:**
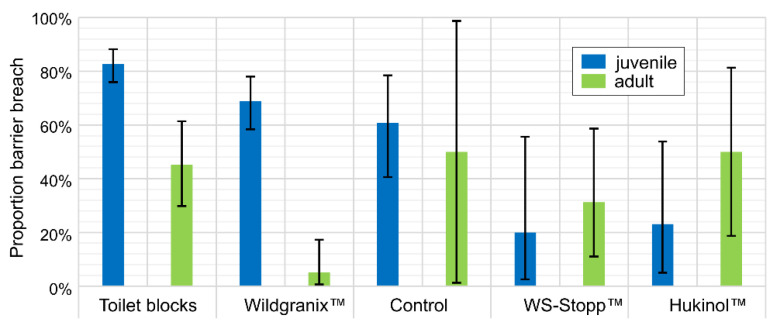
Proportion of barrier breaches with chemical deterrence and 95% confidence intervals.

The effect of the chemical deterrents is shown in Figure 4. Since wild boar abundance had increased markedly at site 1 during the “Wash-out” (Table 1; reasons unknown, maybe there was an alternative feed source—e.g., acorns present in the vicinity only at the beginning of the study), the site could be used as originally planned in the second study phase involving chemical deterrents (i.e., toilet blocks; many wild boar present at site, see Table 2). As compared to the control site, relatively few wild boar passed the barrier with WS-Stopp™ and Hukinol™ (exception: adults) used as deterrents. For the latter, the effect seemed to be more marked for juveniles. On the contrary, the effect of toilet blocks and Wildgranix™ was generally poor concerning juvenile wild boar and significantly less pronounced than on adults, particularly with Wildgranix™, which seemed to be quite effective on adults.

#### 3.2.3. Effect of Deterrents Combined for Age Classes

Since the probability of passing the barrier rack seemed to be different for juveniles and adults in the control as well as for the test sites and the age classes showed up at the sites in different ratios (data not shown), age class was considered a potential confounder [26]. The effect of the deterrents was therefore expressed by producing summary odds ratios of a barrier breach adjusted for the age class according the Mantel–Haenszel procedure [27].

**Figure 5:**
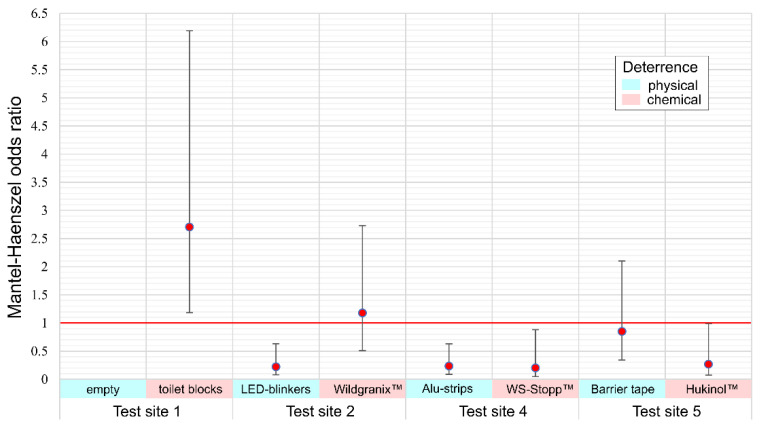
Mantel-Haenszel odds ratios with 95% confidence intervals relative to control.

Summary odds ratios (odds of barrier breach at test site versus control site) are depicted in Figure 5. Odds ratios are more than 2.5 times increased for toilet blocks with the confidence interval not passing through one (odds equal to control) indicating that toilet blocks had a significant effect of attraction rather than deterrence on wild boar. LED blinkers, aluminum strips, WS-Stopp™ and Hukinol™ all had a marked and significant deterring effect with odds ratios under 0.3—i.e., reducing the probability of a barrier breach to about one third. 

## 4. Discussion

Wild boar are notorious for causing damage and losses on grassland, crops and even in forests (in urban areas also gardens). A variety of commercial and non-commercial deterrents has therefore been in use to protect rather large areas over longer periods. Schlageter and Haag-Wackernagel [29] stated that various deterrents are available that claim to be effective in deterring wild boar, but data supporting the claims are scarce or lacking completely. Information on the successful deterrence of wild boar mainly derives from data provided by the manufacturers [30]. 

In contrast to crop protection, a sound deterring effect has to be achieved in carcass guarding only for a small area and a short period (i.e., hours or days). Proper disposal of a carcass should be accomplished within one week after discovery, at the latest. To be on the safe side, deterrents were nonetheless tested for four weeks. Fences (wire only or electrical) were not tested for the following practical reasons. On the one hand, deterrence should be achieved through cheap measures—i.e., devices that are easy to transport and to install, otherwise there would be only a limited advantage of using them over the effort to remove the carcass immediately. On the other hand, barrier breaches concerning fences imply at least a partial destruction of the barrier. The latter would have spoiled the study, even if very short (i.e., impractical) inspection and maintenance intervals could have been implemented.

The study design has some general limitations. First, the wild boar counts at the study sites varied, which may have been due to alternative natural feed resources in the study area—e.g., large numbers of acorns (Table 2). Therefore, the control had to be pooled concerning the two time intervals and no deterrent could be tested on site 1 during interval 1. It turned out to be very difficult to consistently attract wild boar to the study sites. This is why the initial luring phase (Table 1) was expanded from an originally planned duration of 4 weeks to 15 weeks. Unfortunately, the presence of wild boar dropped again, unexpectedly, for some sites when the test phase started. An extension of the test intervals to assure higher wild boar counts was not feasible, since a short-term effect (until proper removal and disposal of carcasses) was the scope of the study. It would have improved the validity of this pilot study if a control for each test site in the respective location had been included. However, this would have implied a sequential design (control phase followed by test phases) and could have led to a bias due to seasonal effects. It was decided to rather eliminate the seasonal effect and test against a control in a different location at the same time. As described above, due to the low wild boar counts at the control site, this approach had to be violated through pooling of the control. If a study design could be implemented in a follow-up study that assures higher wild boar counts, a more sophisticated regression approach should be applied for analysis to account for test site and group size/group composition (juvenile, adult) of wild boar visiting the sites. The following discussion therefore refers to a pilot study set up to assist disease control officials in the selection of deterrents. It should be followed-up by further investigations. 

As physical deterrents, only approaches predominantly relying on optical effects were tested. Yet, except for the LED blinkers, the creation of some noise with movement by wind cannot be ruled out completely. While the LED blinkers and the aluminum strips performed surprisingly well, for both adults and juveniles, the barrier tape failed particularly to deter juvenile wild boar (no barrier breaches of adults, but the sample size was small). This may be explained by the light reflecting or even emitting properties of aluminum strips and LED blinkers versus the limited reflection or partial light absorbance of the barrier tape. Since the deterrents were situated relatively high at the rack to avoid any interference of tactile effects (also for larger wild boar) with the optical ones and potential destruction of the barrier, juveniles with their low visual field might have missed the barrier tape whereas adults spotted the latter more easily. However, the reflected or emitted light of aluminum strips and LED blinkers probably also hit the forest floor and affected the juvenile wild boar. In contrast to our findings, Schlageter and Haag-Wackernagel [30] found LED blinkers to be ineffective, but the authors concede that the red light they used might have been inappropriate as wild boar seem to be unable to discriminate red from gray, but could distinguish blue from gray [31].

Among the chemical deterrents, the toilet blocks showed no deterrent effect. On the contrary, they even seemed to attract, particularly, juvenile wild boar. The scent, though strange, seemed to be somewhat appealing to the wild boar, thwarting the more anecdotal evidence of a deterring potential.

The commercial products WS-Stopp™ and Hukinol™, which contain ingredients that mimic the scent of predators and humans, quite effectively deterred wild boar. However, it is hardly possible to differentiate the effects of the aluminum strips alone and their combination with WS-Stopp™, which was applied to the felt pads of the aforementioned strips according to the manufacturer’s recommendations. The conclusion that there is no effect of the chemical components of the kit (see above) might be misleading. We can only state that there was no additional effect detectable in our study. This might be due to deterrence following a saturation function—i.e., the more effective a deterrent is, the more difficult to achieve is further enhancement. To clarify the effect of the chemical component of WS-Stopp™, a further test with the component applied to a neutral carrier (e.g., cloth) may be considered.

In popular literature [32] both, Hukinol™ and WS-Stopp™ are described to be effective for at least 7–10 days. On the contrary, Schlageter and Haag-Wackernagel [29] found only a minor, non-significant effect in their study on WS-Stopp™. However, the latter study was carried out at a time when the product comprised only component A (see above). Component B was developed later and only then was it included into the commercial kit in response to recommendations from hunters [32]. The new component (B) in combination with component A may therefore explain for the contradicting findings.

Concerning the product Wildgranix™, which did not perform well in this pilot study, it has to be mentioned that due to standardization reasons (see Section 2.1.) the application was not in accordance with the manufacturers recommendations (spread on the ground).

In general, the deterrents seemed to be more effective on juvenile wild boar. This may be due to the lack of experience of the juveniles on the one hand. On the other hand, the sociology of wild boar implies that juveniles act explorative, while the adults safeguard (i.e., outside the luring area). Particularly for Hukinol™ and WS-Stopp™, this general pattern tended to be inverted in our study, although the results were not statistically significant. One might speculate that the intense scent of predators mimicked by the products effectively addresses innate instincts of juvenile wild boar.

It has to be borne in mind that wild boar carcasses as the entity of attraction were substituted by small luring sites with baiting automats feeding corn in our study. This was done for the sake of standardization, in particular to avoid potentially inherent initial differences in the carcass attractiveness and differences due to varying courses of decomposition and scavenger activities. Moreover, corn is supposed to be more of a challenge to a deterrent than carcasses, which are only moderately attractive to wild boar [8]. Consequently, it may be reasonable to assume an even more pronounced effect of the deterrents identified as suitable candidates in the presented study when applied to carcasses.

Due to the aforementioned limitations, an in-depth investigation into the efficiency of the deterrents identified as promising by this pilot study is recommended.

## 5. Conclusions

This study showed that wild boar can be deterred from attractants (maize) through deterrents that are reasonable and easy to use. If ASF occurs in wild boar, suitable deterrents may be carried by rangers in carcass searches and used to keep the animals off dead conspecifics. Even if a total deterrence is impossible, deterrents may be applied as a provisional measure, if immediate removal and safe disposal of a carcass is not feasible, and thus reduce the risk of disease transmission in the natural habitat of wild boar.

## Figures and Tables

**Table 1:** Study plan for the five study sites (Figure 1).

		Week		
Study Site	1–15 ^1^	16–19 ^2^	20–23 ^1^	24–27 ^2^
3-Control	Initial luring	---	“Wash-out”	---
1-Test	Initial luring	---	“Wash-out”	Toilet blocks
2-Test	Initial luring	LED blinkers	“Wash-out”	Wildgranix™
4-Test	Initial luring	Alu strips	“Wash-out”	WS-Stopp™
5-Test	Initial luring	Barrier tape	“Wash-out”	Hukinol™

Luring was carried out on all sites and throughout the study (week 1–27) with—^1^ widespread baiting, ^2^ narrow baiting only inside “luring area” (Figure 2).

**Table 2:** Wild boar count at the study sites during test phases.

	Week/Deterrent/Count	
Study Site	16–19	24–27
3-Control	---^1^ (20/0) ^2^	--- (10/2)
1-Test	--- (not recorded)	Toilet blocks (162/42)
2-Test	LED blinkers (30/85)	Wildgranix™ (93/39)
4-Test	Alu strips (38/55)	WS-Stopp™ (10/16)
5-Test	Barrier tape (51/7)	Hukinol™ (13/10)

^1^ No deterrent applied, ^2^ (Juvenile/Adult).
